# Inter-Reader Agreement of Diffusion-Weighted Magnetic Resonance Imaging for Breast Cancer Detection: A Multi-Reader Retrospective Study

**DOI:** 10.3390/cancers13081978

**Published:** 2021-04-20

**Authors:** Filippo Pesapane, Anna Rotili, Silvia Penco, Marta Montesano, Giorgio Maria Agazzi, Valeria Dominelli, Chiara Trentin, Maria Pizzamiglio, Enrico Cassano

**Affiliations:** 1 Radiology Department, Breast Imaging Division, IEO European Institute of Oncology IRCCS, 20141 Milan, Italy; anna.rotili@ieo.it (A.R.); silvia.penco@ieo.it (S.P.); marta.montesano@ieo.it (M.M.); valeria.dominelli@ieo.it (V.D.); chiara.trentin@ieo.it (C.T.); maria.pizzamiglio@ieo.it (M.P.); enrico.cassano@ieo.it (E.C.); 2 Department of Radiology, University of Brescia, 25123 Brescia, Italy; g.agazzi001@unibs.it

**Keywords:** breast cancer, radiology, diffusion-weighted imaging, oncology, agreement, reproducibility

## Abstract

**Simple Summary:**

The role of magnetic resonance imaging (MRI) in breast cancer has expanded in the last decade, and studies have demonstrated good sensitivity and specificity of diffusion-weighted imaging (DWI), a functional imaging technique reflecting water diffusion properties in tissues. However, clear results about the reproducibility of DWI are still missing. To utilize DWI as a reliable stand-alone technique for breast cancer detection, the inter-reader agreement of the measurement must be assessed. Accordingly, in this study, we assess the inter-reader reproducibility to retrospectively evaluate the agreement of breast cancer detection using DWI as a stand-alone technique. As our results show a good agreement only in expert readers, the assumption that a breast MRI based only on qualitative analysis of DWI, with fewer variables, may be easier for a non-expert reader to learn seems disproved, and future prospective studies should assess the right time for appropriate training for radiologists to investigate the potential role of DWI as a stand-alone method for un-enhanced breast MRI.

**Abstract:**

Purpose: In order to evaluate the use of un-enhanced magnetic resonance imaging (MRI) for detecting breast cancer, we evaluated the accuracy and the agreement of diffusion-weighted imaging (DWI) through the inter-reader reproducibility between expert and non-expert readers. Material and Methods: Consecutive breast MRI performed in a single centre were retrospectively evaluated by four radiologists with different levels of experience. The per-breast standard of reference was the histological diagnosis from needle biopsy or surgical excision, or at least one-year negative follow-up on imaging. The agreement across readers (by inter-reader reproducibility) was examined for each breast examined using Cohen’s and Fleiss’ kappa (κ) statistics. The Wald test was used to test the difference in inter-reader agreement between expert and non-expert readers. Results: Of 1131 examinations, according to our inclusion and exclusion criteria, 382 women were included (49.5 ± 12 years old), 40 of them with unilateral mastectomy, totaling 724 breasts. Overall inter-reader reproducibility was substantial (κ = 0.74) for expert readers and poor (κ = 0.37) for non- expert readers. Pairwise agreement between expert readers and non-expert readers was moderate (κ = 0.60) and showed a statistically superior agreement of the expert readers over the non-expert readers (*p* = 0.003). Conclusions: DWI showed substantial inter-reader reproducibility among expert-level readers. Pairwise comparison showed superior agreement of the expert readers over the non-expert readers, with the expert readers having higher inter-reader reproducibility than the non-expert readers. These findings open new perspectives for prospective studies investigating the actual role of DWI as a stand-alone method for un-enhanced breast MRI.

## 1. Introduction

The role of magnetic resonance imaging (MRI) in breast cancer has expanded in the last decade, and currently includes tumor detection and characterization, screening in high-risk patients, image guidance for biopsy, and treatment response scenarios [1,2,3,4,5,6,7,8,9].

The backbone of MRI techniques for assessing breast cancer is the dynamic contrast-enhanced (DCE) acquisition, which characterizes tissue vascularity, and was demonstrated to be the most reliable tool for assessing the loco-regional extent of breast cancer [10]. However, growing evidence supports diffusion-weighted imaging (DWI), a functional imaging technique reflecting water diffusion properties in tissue, as a supplemental, or even alternative, technique to DCE. Such an innovation, if it were used instead of DCE, could minimize costs, reduce the time spent, and therefore improve patients’ and physicians’ compliance with MRI. Moreover, the lack of intravenous injections of gadolinium-based contrast agents (GBCA) could avoid the gadolinium retention in the brain and other tissues, especially in patients with high risk of breast cancer who undergo repeated breast MRI [5,7,11,12,13].

Several studies have demonstrated good sensitivity and specificity in DWI combining b-value sequences and the apparent diffusion coefficient (ADC) map in the detection of breast cancer [14].

We have recently shown, using a retrospective approach, 93% sensitivity and 88% specificity of DWI as a stand-alone screening method for breast cancer [7].

However, our results, like others, were obtained by expert readers, and this has been a potential barrier to the expansion of the technique to a widespread use of breast MRI [12]. Concerns remain about DWI reproducibility, with studies showing poor to moderate agreement [15,16].

Notably, it has recently been suggested that radiologists who are already competent at reading mammograms can achieve similar levels of accuracy of interpretation of the abbreviated MRI protocol proposed by Kuhl C.K. et al. (consisting of first postcontrast subtracted images and maximum-intensity projection only) [17] to that of expert breast MRI readers [18].

Similarly, in order to utilize DWI as a stand-alone, reliable technique for breast cancer detection, the inter-reader agreement of the measurement must be assessed. Agreement between measurements refers to the degree of concordance between two (or more) sets of measurements, and statistical methods to test agreement are used to assess the inter-rater variability or to decide whether one technique for measuring a variable can substitute another [19]. Concerning variability, the terms ‘reproducibility’ and ‘repeatability’ are used with varying degrees of consistency in the medical literature [20]. While repeatability of measurements refers to the variation in repeat measurements provide by the same subject under identical conditions, reproducibility refers to the variation in measurements provided by a subject under changing conditions [21]. In our study, we wanted to assess the inter-reader reproducibility to retrospectively evaluate the agreement of breast cancer detection using DWI as a stand-alone technique.

## 2. Materials and Methods

### 2.1. Patient Population

Consecutive women referred for breast MRI at a referral breast cancer care between January and September 2016 were retrospectively evaluated.

All patients were over the age of 18 years, not pregnant or breastfeeding, and had no contraindications to MRI. Table 1 reports the indication criteria for MRI in our population. 

Standards of reference were the histological analysis through biopsy or surgery or ≥ 1 year of clinical and radiological follow-up. Table 2 shows inclusion and exclusion criteria.

**Figure 1:**
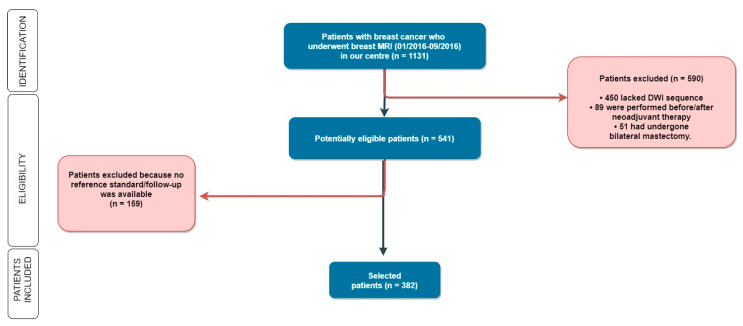
Out of 1131 women enrolled in our study, we selected 382 patients according to our inclusion and exclusion criteria. MRI: Magnetic Resonance Imaging; DWI: Diffusion Weighted Imaging.

As shown in Figure 1, patients were subsequently excluded if the DWI sequence was not available (i.e., when DWI was not included in the imaging protocol), in the case of no follow-up, when the surgery was performed in other hospitals, or in the case that patients underwent neoadjuvant chemotherapy because the size of locally advanced or relatively large lesions could be easy to detect, possibly representing a bias in our agreement assessment.

### 2.2. MRI Technique

The MR examinations were performed with the patient in prone position using a 1.5 T scanner (Optima MR450w, General Electric Medical Systems) equipped with a 34 mT/m gradient and a dedicated eight-channel breast coil. The MRI standard protocol at our institution includes: a three-plane localizer, axial FSE T2 weighted images, axial DWI with the relative apparent diffusion coefficient (ADC) maps, dynamic series performed once before and four times after intra-venous administration of 0.1 mmol/kg of a gadolinium-chelate at 90 s, post-processing subtraction, and maximal intensity projection (MIP) images.

The technical parameters of the two-dimensional echo-planar spin-echo DWI sequence were as follows: time of repetition 3836 ms; time of echo 64 ms; inversion time 190 ms; flip angle 90°; pixel bandwidth 1953.12; b-values 0 and 1000 s/mm^2^; spatial resolution 2 × 3.6 × 5 mm (gap interslice 0.2 mm); number of excitations 1. Acquisition time varied from 3 min and 31 s to 6 min 22 s, depending on breast size.

### 2.3. Image Analysis and Readers’ Characteristics

Four readers from two different institutions with different levels of experience qualitatively read breast MRI and blindly assessed DWI images with relative ADC maps for each breast. Given the aim of the study, the contrast–enhanced sequences were not evaluated and, therefore, readers had no access to DCE sequences, subtracted images, kinetic curves, or MIP images. Moreover, all MRIs were anonymized, and readers were blinded to the clinical history of the study subjects including prior MR, mammography, and ultrasound (US) examinations.

Reader 1 (BLINDED, named Expert_1) had 10 years of experience (approx. 8500 examinations); reader 2 (BLINDED, named Expert_2) had 7 years of experience (approx. 2500 examinations); reader 3 (BLINDED, named NonExpert_1) had 3 years of experience (approx. 1000 examinations); and reader 4 (BLINDED, named NonExpert_2) had 9 months of experience (approx. 250 examinations). Readers 1 and 2 were considered as expert readers, while readers 3 and 4 were considered as non-expert readers. Both expert and non-expert readers had some (minor) experience in non-breast MRI including DWI/ADC sequences (particularly multiparametric prostate MRI).

**Figure 2:**
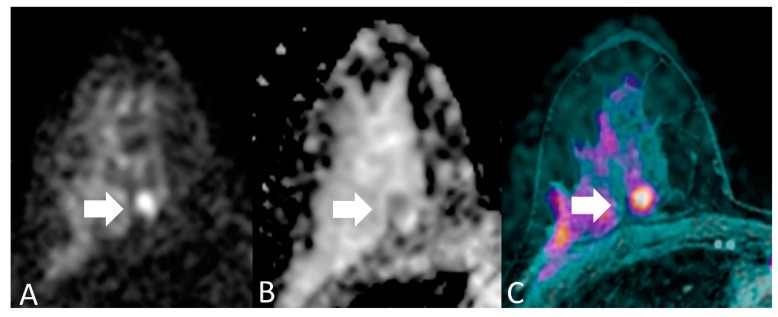
A 46-year-old woman with medium degree of differentiation ductal carcinoma of the right breast, in a deep position (arrows). (**A**) DWI (b = 1000 s/mm^2^) shows a focal with hyperintensity of the signal; (**B**) ADC map shows a focal hypointensity in the same location; (**C**) T1-weighted sequence with fat saturation, post-contrast, (not considered in the present study) reveals the presence of a suspicious lesion in the same site. On histopathological examination, the lesion had a diameter of < 10 mm. This is an example of discrepancy between experienced readers’ (true positive) and non-experienced readers’ (false negative) readers results.

For each patient, readers evaluated each breast separately. Breast MRI assessment based on DWI includes both qualitative interpretation of diffusion-weighted images for lesion detection and quantitative measures of ADC for lesion characterization. Qualitatively, areas of restricted diffusion will have higher signal intensity on DWI and lower signal intensity on ADC map images. The essential concept behind detecting malignancy with quantitative diffusion imaging is that breast cancer has significantly lower ADCs than benign breast lesions or normal tissue, due to the relatively increased tumor cellularity which restricts diffusion, manifested by a bright signal on DWI and dark signal on a corresponding ADC map [7,16]. We considered an imaging finding as a positive case (namely, a lesion with imaging features suspicious for breast cancer) when it showed a focal with hyperintensity of the signal at DWI (b = 1000 s/mm^2^) and a hypointensity to the ADC map (Figure 2) with a threshold value of ADC of 1.23 × 10^−3^ mm^2^.

Although the per-breast evaluation was performed according to the Breast imaging-reporting and data system (BI-RADS) diagnostic classification [22], for the final unenhanced assessment, BI-RADS 0 category was not permitted and BI-RADS 6 was not possible (blinded reading). Thus, the scale was dichotomized in two categories: negative (BIRADS 1, 2, and 3) versus positive (BI-RADS 4 and 5). In positive cases, the readers recorded the localization and diameters of the main lesion to ensure that they had identified the same target lesion.

Patient age, dimensions, and histopathological diagnosis of the main lesion detected on MRI were electronically reviewed for each patient.

The per-breast standard of reference was the histological diagnosis from needle biopsy or surgical excision, or at least one-year negative follow-up on imaging.

### 2.4. Statistical Analysis

Descriptive statistics are reported as mean ± standard deviation (SD) or median and interquartile range (IQR) according to normal/near-normal or non-normal data distribution.

Per-breast sensitivity, specificity, and accuracy were calculated for each reader. Point-estimates were given with a 95% confidence interval (CI) according to the binomial distribution. 

The prevalence of ductal carcinoma in situ (DCIS) and non-mass enhancement among false negatives and true positives was compared through χ^2^ test.

The agreement was examined across readers for each breast examined by MRI, and was assessed through the calculation of inter-reader reproducibility using Cohen’s and Fleiss’ kappa (κ) statistics. The values of κ were considered as follows: 0–0.20, slight agreement; 0.21–0.40, fair agreement; 0.41–0.60, moderate agreement; 0.61–0.80, substantial agreement; 0.81–1, almost perfect agreement [23,24].

Particularly, Cohen’s κ was used in the case of pairwise reader comparison (inter-reader or paired inter-reader stratified by experience) while Fleiss’ kappa was used in the case of four-reader comparison (overall inter-reader reproducibility).

The Wald test was used to test the difference in inter-reader agreement between expert and non-expert readers [25], and *p*-value corresponds to two-sided tests, with *p* < 0.05 considered to represent a significant difference.

Statistical calculations were performed using R 4.0 software [26].

## 3. Results

Of 1131 examinations, according to our inclusion and exclusion criteria (Table 2), we selected a total of 382 women aged 49.5 ± 12 years (mean ± SD; range 20–80 years), 40 of them (10.5%) with unilateral mastectomy, totaling 724 breasts available for analysis (Figure 2).

Per-patient cancer prevalence was 96/382 (25.1%), per-breast cancer prevalence 96/724 (13.3%). There were 60 (63%) invasive ductal, nine (9%) invasive lobular, nine (9%) invasive ductal–lobular, eight (8%) DCIS, one (1%) invasive ductal or papillary, one (1%) mucinous, one (1%) tubular, one (1%) primary small-cell neuroendocrine, and six (6%) not otherwise specified breast carcinomas.

Median size at pathology was 18 mm (IQR 25–11 mm). Follow-up ranged from 12 to 39 months (mean ± SD, 20 ± 4 months).

Figure 2 shows an example of breast MRI of one woman included in our patient population.

Table 3 and Table 4 show the diagnostic performance of stand-alone DWI in the detection of a lesion and the sensitivity of the breast through dimensional stratification of lesions, respectively.

Overall inter-reader reproducibility for all readers was moderate (κ = 0.56), while it was substantial (κ = 0.74) for expert readers, and poor (κ = 0.37) for non- expert readers.

Expert readers showed inter-reader reproducibility occurring in 90.7% (656/724) of breasts while low-experience readers only in 50.1% (368/724).

Pairwise agreement (in terms of inter-reader reproducibility) between expert readers and non-expert readers was moderate (κ = 0.60) and showed a statistically superior agreement of the expert readers over the non-expert readers (*p* = 0.003).

**Figure 3:**
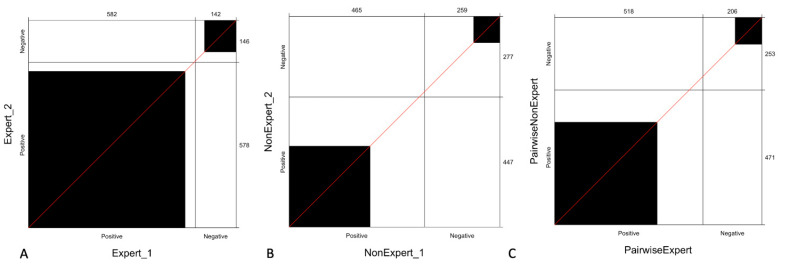
Agreement of readers stratified by experience: the panel shows the observed agreement between expert readers (**A**), non-expert readers (**B**) and pairwise observed agreement between expert and non-expert readers (**C**). The black square area indicates the exact observed agreement between the two operators while the white remaining area indicates the disagreement. We can observe that the agreement between expert readers is higher (**A**) compared to that observed with non-expert (**B**) or pairwise expert and non-expert readers (**C**).

Figure 3 shows the observed agreement between expert readers, non-expert readers, and pairwise agreement stratified by experience, which reveals that the agreement between expert readers is higher compared to that observed with non-expert readers and even with pairwise expert and non-expert readers (C).

Using a single consensus score between the two expert readers, the inter-reader reproducibility between the consensus and reader 4 was poor (κ = 0.24), while the inter-reader reproducibility between the consensus of the expert readers and reader 3 was moderate (κ = 0.58).

**Figure 4:**
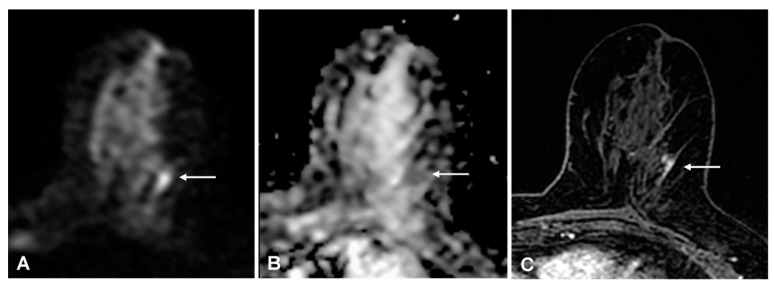
(**A**) 54-year-old woman with a 15-mm invasive ductal carcinoma: true-positive finding on diffusion-weighted imaging (DWI) that was detected by expert readers only. A, DWI (b = 1000 s/mm^2^) shows a mild linear hyperintense lesion in the upper outer portion of the left breast (arrow). (**B**) apparent diffusion coefficient-map shows corresponding homogeneous hypo-intensity. (**C**) for comparison, the axial T1-weighted fat-sat contrast-enhanced image shows focal non-mass enhancement in the same location (arrow).

With the experts’ single consensus score, the false-positive rate was 3% (23/609) and the false-negative rate was 7% (7/96): four low-grade invasive cancers (three ductal and one tubular histological subtype), and three ductal carcinomas in situ (DCIS) were missed; 4/89 (4%) true positive findings were DCIS (*p* < 0.001); 6/7(86%) false-negative and 17/89 (19%) true positive findings were non-mass enhancements (*p* < 0.001). All three false-negative DCIS were non-mass enhancement. Expert readers rightly detected 5/8 (63%) DCIS and 16/20 (80%) non-mass enhancement malignant lesions, while non-expert readers detected 3/8 (62%) DCIS and 11/20 (55%) non-mass enhancement malignant lesions (Figure 4 shows an example with discrepant findings between experts and non-experts).

## 4. Discussion

The steadily increasing demand for breast MRI has led to concerns regarding the lack of access to MRI itself, which is expensive and time-consuming, as well as concerns regarding the potential side-effects of GBCA (e.g., gadolinium toxicity and nephrogenic systemic fibrosis) and its tissue retention [5,7,9,11,12,13,27,28].

Solutions must enhance operational benefits without compromising diagnostic performance or decreasing reader reproducibility. One approach may be the implementation of MRI without the use of GBCA, but only if certain prerequisites are ensured, for example high-quality imaging, interpretation quality, and availability of patient recall or on-table monitoring.

Diffusion-weighted imaging is an MRI technique that measures the random Brownian motion of water molecules within a tissue, giving functional information on tissue microstructure without the need for intravenous GBCA [28]. Breast cancers present an increase in cell density and restriction of water diffusion, showing a higher signal on DWI and a lower signal on ADC map than benign lesions and normal tissue, allowing for lesion differentiation with pooled sensitivities ranging 84–91% and specificities ranging 75–84%, as shown by recent meta-analyses [29,30]. Notably, in a recent study performed in our centre based on a similar population, we demonstrated that DWI showed a 93% sensitivity and 88% specificity, with 71% sensitivity for cancers ≤ 10 mm, indicating potential for DWI as a stand-alone screening method [7].

Moreover, DWI showed similar performance to that usually reported for a full MRI including DCE sequences in other recent studies [16,31,32]. A recent survey from the European Society of Breast Imaging reported 60% of responders to consistently apply DWI in clinical practice [33], but there is a need for studies where clinical decisions are based upon breast MRI without DCE, which must define clinical and operational benefits and identify which patient groups can be scanned successfully without using GBCA.

Although recent studies showed that DWI is sensitive to tissue microstructure and cellularity, and provides quantitative information that can be used for lesion characterization, the lack of standardization of DWI protocols has caused a huge variability in DWI/ADC estimation and interpretation methods across clinical sites [34]. To determine the real value of DWI as a stand-alone technique in breast MRI, a standardized acquisition protocol and interpretation approach is demanded. Therefore, an International Breast DWI Working Group was established by the European Society of Breast Radiology (EUSOBI) to support implementation of DWI in breast MRI through standardized and reproducible acquisitions, and to promote its use in diagnostic and prognostic clinical practice through its adoption as an integral part of standardized guidelines like the BI-RADS [34].

Currently, standard DWI calculates ADC values using Gaussian monoexponentially modeling, which has shown different optimal ADC cutoffs in the literature in differentiating benign from malignant breast lesions [35]. The EUSOBI DWI working group found consensus on a minimal set of acquisition parameters to be met in clinical practice such as two number of values (namely the 0 s/mm^2^ and the 800 s/mm^2^) and a slice thickness < 4 mm [34]. Adherence to these minimal requirements should improve the comparison of ADC values from site to site, which is an important step towards the generalizability required to eventually incorporate ADC quantification into BI-RADS.

As our breast MRI protocol was optimized in 2016 and has not yet been updated with recent EUSOBI indications [34], we used as b values the 0 s/mm^2^ and the 1000 s/mm^2^ (instead of 800 s/mm^2^). Accordingly, using a 2 d ROI for calculating the ADC, we chose a threshold for malignancy of 1.23 × 10^−3^ mm^2^/sec at b = 1000 sec/mm^2^ as a meta-analysis of 12 articles recommended [36].

Although technically challenging, DWI protocol standardization between different systems has been achieved in many organs and this encourages further studies in DWI [34,35]; our large retrospective single-centre study fits into this line of research. Through the analyses of inter-reader reproducibility, we tested the agreement of DWI as a tool for breast cancer detection by MRI without the use of intravenous GBCA.

In our results, we reported a wide range of agreement across all readers, and non-expert readers showed an overall poor reproducibility, suggesting that readers should have at least 3 years of experience to evaluate DWI as a stand-alone sequence of breast MRI. Particularly, the inter-reader reproducibility results were poor for non-expert readers, while it was substantial for expert readers. Such results seem to disprove the assumption that a breast MRI based only on DWI/ADC, with fewer variables than a contrast enhanced breast MRI, may be easier for a non-expert reader to learn.

Moreover, we compared the consensus between expert readers and the consensus between non-expert readers. Expert readers showed a significantly higher inter-reader reproducibility than non-expert readers (*p* = 0.003). The low agreement of non-expert readers suggests the unsuitability of a breast MRI based only on DWI by radiologists who do not have adequate experience.

Finally, using a consensus between the two expert readers, the inter-reader reproducibility between that consensus and NonExpert_2 (9 months of experience, namely the reader with the lowest experience) was poor, while the inter-reader reproducibility between the consensus of the expert readers and NonExpert_1 (3 years of experience) was moderate.

This suggests a learning curve that requires at least 3 years of experience to appropriately read a breast MRI based on DWI. Nevertheless, it is not possible to draw conclusions from this observation because such differences among readers may be due to individual predisposition and personal skills. Particularly in relation to the non-expert readers, comprising only two participants, the disagreement may be caused by individual ability and not by different levels of expertise. This is one of the main limitations of this study.

Other limitations, beyond the retrospective design of the study, include the following: (1) the relatively high lesion size could have affected the visibility of breast cancers. Nevertheless, the cancers median size of 18 mm was still in the context of early breast cancer (which it means that the cancer is growing but it is still contained in the breast or growth has only extended to the nearby lymph nodes); (2) many MR exams were exluded as DWI was not routinely performed in the original protocol (at the time of the patient’s enrollment), although, to the best of our knowledge, this is one of the largest patient cohorts investigated for exploring DWI performance for breast cancer detection [35]; (3) the follow-up time was rather short in some of the patients, partially because patients prefer to be followed up near home at other institutions once staging and treatment have been set up in our tertiary cancer care.

Despite the limits of our study, we believe that a solution to improve the agreement may be the creation of a specific scoring template for breast MRI based only on DWI and ADC-map to decrease the subjectivity involved with interpreting DWI signals. Assessment of in-house agreement at individual breast MRI centres for purposes of quality control may further improve diagnostic precision.

We should finally note that DWI sequences are undergoing a continuous technical refinement. New radiofrequency coil design, advanced techniques and improved shimming may help to overcome some of the technical obstacles of achieving high-quality breast DWI [34,35,36,37,38]. A higher spatial resolution allowing for a superior lesion conspicuity and morphology evaluation may improve inter-reader reproducibility and may favor an improvement in, and more confident utilization of, DWI as well.

## 5. Conclusions

In conclusion, our study found DWI to have poor to substantial inter-reader reproducibility among non-expert- to expert-level readers, while pairwise comparison showed superior agreement of the expert readers over the non-expert readers, with expert readers having higher inter-reader reproducibility than non-expert readers.

These findings have implications for the interpretation of agreeability and performance in multi-reader studies, and they open the way to prospective studies investigating the potential role of DWI as a stand-alone method for un-enhanced breast MRI.

## Figures and Tables

**Table 1:** Indications for MRI in our population.

Indication	Number of Patients	%
Screening in high-risk patients	156	41
Problem solving for inconclusive mammogram or US examination	74	20
Preoperative staging	67	18
Follow-up in previous breast cancer	63	16
Nipple discharge	7	2
Needle biopsy showing borderline lesions (B3)	6	1
CUP syndrome	5	1
Post-operative positive margins	4	1
Total	382	100

US: ultrasound; CUP: cancer of unknown primary.

**Table 2:** Inclusion and exclusion criteria. MRI: Magnetic Resonance Imaging.

Inclusion Criteria:	Exclusion Criteria:
≥18 years old women	pregnancy or breastfeeding
at least one year of clinical and radiological follow-up or histological analysis through biopsy or surgery	patients who underwent surgery or follow-up in other hospitals/centres
written informed consent for MRI must be signed and dated by both the patient and the radiologist	patients undergoing neoadjuvant chemotherapy
	common contraindications to MRI (presence of pacemaker non-MRI-safety or claustrophobia)

**Table 3:** Per-breast diagnostic performance of stand-alone DWI in 724 breasts in 382 women. Data in parentheses represent percentages and their 95% confidence intervals.

Performance Index	Expert_1	Expert_2	NonExpert_1	NonExpert_2
Sensitivity	89/96 (93%, 86–96%)	83/96 (87%, 78–93%)	81/96 (84%, 82–93%)	77/96 (80%, 76–89%)
Specificity	562/609 (93%, 90–94%)	538/609 (88%, 86–91%)	526/609 (86%, 83–89%)	511/609 (83%, 81–879%)
Accuracy	645/724 (89%, 87–92%)	698/724 (96%, 92–98%)	640/724 (88%, 86–92%)	615/724 (84%, 82–98%)

**Table 4:** Per-lesion diagnostic performance (in terms of sensitivity) of DWI of a double reading of expert according to different tumor size.

Tumor Size	Sensitivity of Double Reading of Expert_1 and Expert_2)	*p*-Value
≤ 10 mm	25/33(75%, 52–86%)	0.010
> 10–20 mm	44/48 (92%, 82–99%)	
> 20 mm	36/39 (92%, 78–98%)	

Data in parentheses represent percentages and their 95% confidence intervals. *DWI* = Diffusion-weighted imaging. Chi-square test was used. At post-hoc analysis: ≤10 mm versus >10–20 mm, *p* = 0.009; ≤10 mm versus >20 mm, *p* = 0.028; > 10–20 mm versus > 20 mm, *p* = 0.776.

## Data Availability

The data presented in this study are available on request from the corresponding author. The data are not publicly available due to privacy reason, according to GDPR.
